# Vascular Aging across the Menopause Transition in Healthy Women

**DOI:** 10.1155/2014/204390

**Published:** 2014-07-17

**Authors:** Kerrie L. Moreau, Kerry L. Hildreth

**Affiliations:** Division of Geriatric Medicine, University of Colorado Denver, Building L15 Rm 8111, 12631 East 17th Avenue, P.O. Box 6511, Aurora, CO 80045, USA

## Abstract

Vascular aging, featuring endothelial dysfunction and large artery stiffening, is a major risk factor for developing cardiovascular disease (CVD). In women, vascular aging appears to be accelerated during the menopause transition, particularly around the late perimenopausal period, presumably related to declines in ovarian function and estrogen levels. The mechanisms underlying endothelial dysfunction and large artery stiffening with the menopause transition are not completely understood. Oxidative stress and the proinflammatory cytokine tumor necrosis factor-α contribute to endothelial dysfunction and large artery stiffening in estrogen-deficient postmenopausal women. Habitual endurance exercise attenuates the age-related increase in large artery stiffness in estrogen-deficient postmenopausal women and can reverse arterial stiffening to premenopausal levels in estrogen-replete postmenopausal women. In contrast, estrogen status appears to play a key permissive role in the adaptive response of the endothelium to habitual endurance exercise in that endothelial improvements are absent in estrogen-deficient women but present in estrogen-replete women. We review here the current state of knowledge on the biological defects underlying vascular aging across the menopause transition, with particular focus on potential mechanisms, the role of habitual exercise in preserving vascular health, and key areas for future research.

## 1. Introduction

Despite significant declines in cardiovascular disease (CVD) mortality, CVD is still the leading cause of death in adults [1]. Vascular aging, featuring endothelial dysfunction and large artery stiffening, is a major risk factor for the development of CVD, in that it combines with other known risk factors to create an age-disease interaction [2]. In women, vascular aging is unique in that adverse changes in CVD risk factors (e.g., blood pressure, lipids, and adiposity) occur during a time of profound changes in the hormonal environment as women transition through menopause. The acceleration of age-associated declines in vascular function in women after menopause suggests that menopause may be a triggering event that leads to increased vascular vulnerability as women age. Thus, understanding the underlying biological defects associated with vascular aging across the menopause transition is important for the development of strategies to maintain vascular health and decrease CVD mortality.

This review will focus on some of the work that we have done on the modulatory influence of sex hormone deficiency on vascular aging in healthy women. We will discuss the underlying mechanisms that we have studied to date and the role of habitual endurance exercise in promoting healthy vascular aging in women. Finally, we also discuss gaps in knowledge and identify key areas for future research to advance women’s health across the menopause transition.

## 2. Vascular Endothelial Dysfunction

**Figure 1:**
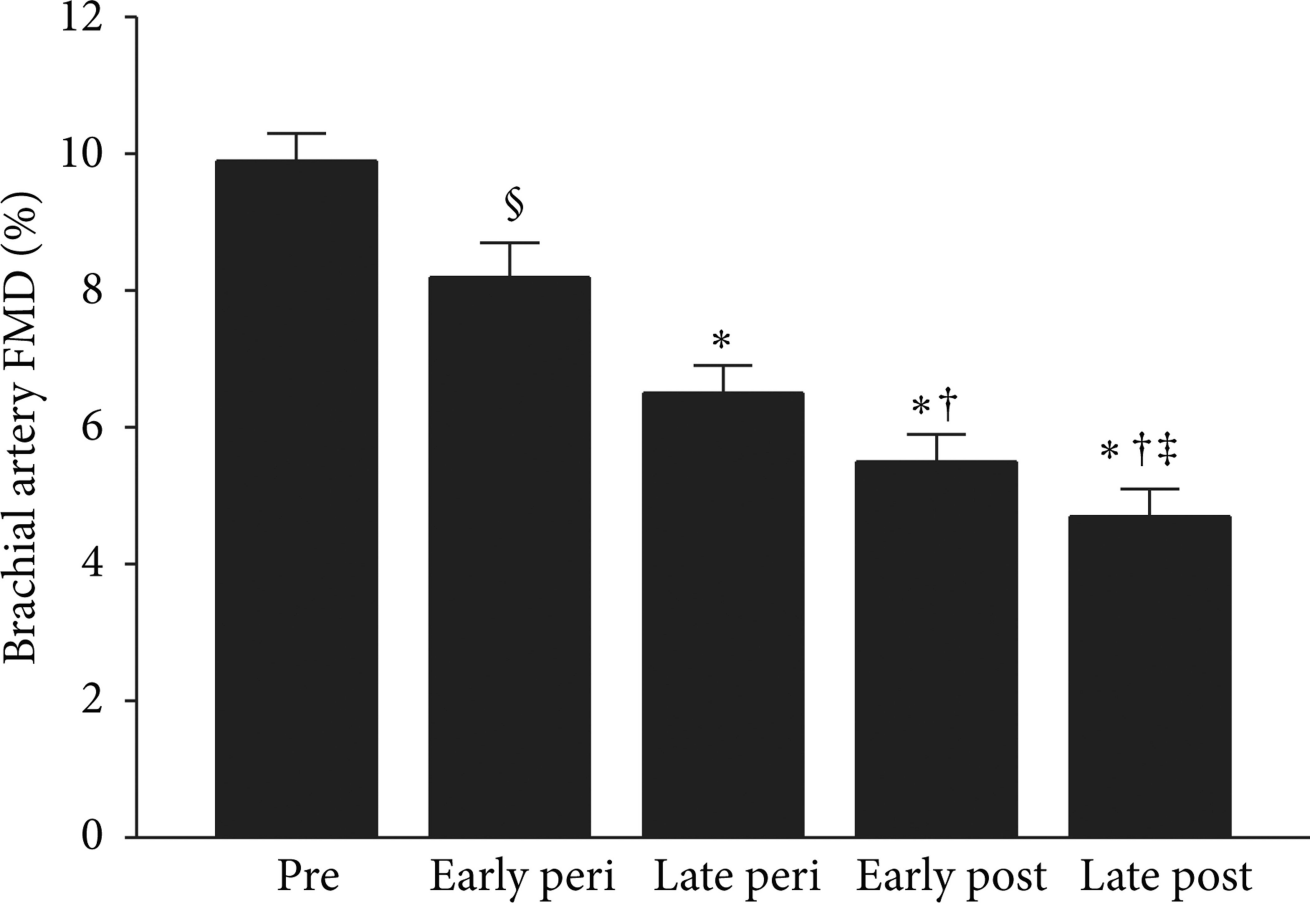
Brachial artery EDV declines across the stages of the menopause transition. The effect of menopause stage was independent of aging and CVD risk factors. * *P* < 0.001 and ^§^ *P* = 0.03 versus premenopausal women; ^†^ *P* < 0.001 versus early perimenopausal; ^‡^ *P* < 0.001 versus late perimenopausal. Peri = perimenopausal; post = postmenopausal. From Moreau et al. [3].

Endothelial dysfunction, characterized by reduced endot-helial-dependent vasodilation (EDV), is a significant predictor of cardiovascular events [10]. Because the vascular endothelium plays a key role in the maintenance of vascular health [11], the loss of normal endothelial function is thought to be a critical step in the initiation and progression of atherosclerosis [2]. Classical studies conducted in the 1990’s demonstrate that aging is associated with a progressive decline in EDV of large conduit arteries (measured via brachial artery flow-mediated dilation (FMD)) and of resistance vessels (forearm blood flow response to intra-arterial acetylcholine infusion) in healthy adults [12, 13]. The rate of decline was different between men and women. Men demonstrated a gradual decline after the fourth decade; in women, declines were delayed approximately one decade but accelerated after menopause [12, 13]. These data suggested a protective effect of estrogen on endothelial function in women. Because the previous studies only included premenopausal and postmenopausal women, we examined whether hormonal changes during the perimenopausal years influenced the rate of decline in endothelial function in women. We demonstrated that the decline in EDV(measured via brachial artery FMD) actually begins during the early perimenopausal period but was more pronounced during the late perimenopausal period. Relative to premenopausal women, early perimenopausal women had a 17% decline in brachial artery FMD; in late perimenopausal women of similar age, this decline was doubled (∼35%). Moreover, the decline in EDV worsened during the postmenopausal period (see Figure 1) [3]. The decline in EDV across menopausal stages was independent of age and traditional CVD risk factors [3]. These findings suggested that ovarian hormone levels in the early perimenopausal period may be sufficient to provide some level of endothelial protection and that declines in ovarian function and estrogen levels in the late perimenopausal transition initiate the rapid deterioration in endothelial function that worsens with prolonged estrogen deficiency. Importantly, we have demonstrated that short-term estrogen replacement therapy can improve EDV by ∼50–55% [5, 9, 14]. However, the estrogen replacement does not fully restore EDV to youthful premenopausal levels. The reasons for this are unclear but may be related to the length of time of estrogen deprivation and/or age-associated phenotypic changes in the vascular endothelium that diminish endothelial signaling or responsiveness to estrogen [15, 16].

### 2.1. Mechanisms Underlying Endothelial Dysfunction across the Menopause Transition

The mechanisms mediating the decline in EDV across the stages of the menopause transition are not completely understood. A decrease in the bioavailability of the vasodilator nitric oxide (NO) is considered a key factor contributing to the impairment in EDV with estrogen deficiency and aging. Nitric oxide is produced by the interaction of L-arginine and endothelial nitric oxide synthase (eNOS) and upon its release activates guanylate cyclase, subsequently producing cyclic-GMP and causing smooth muscle cell vasodilation. Thus, any defects in NO synthesis, and/or enhanced breakdown of NO, could influence the bioavailability of NO.

### 2.2. Estrogen Receptor Signaling

**Figure 2:**
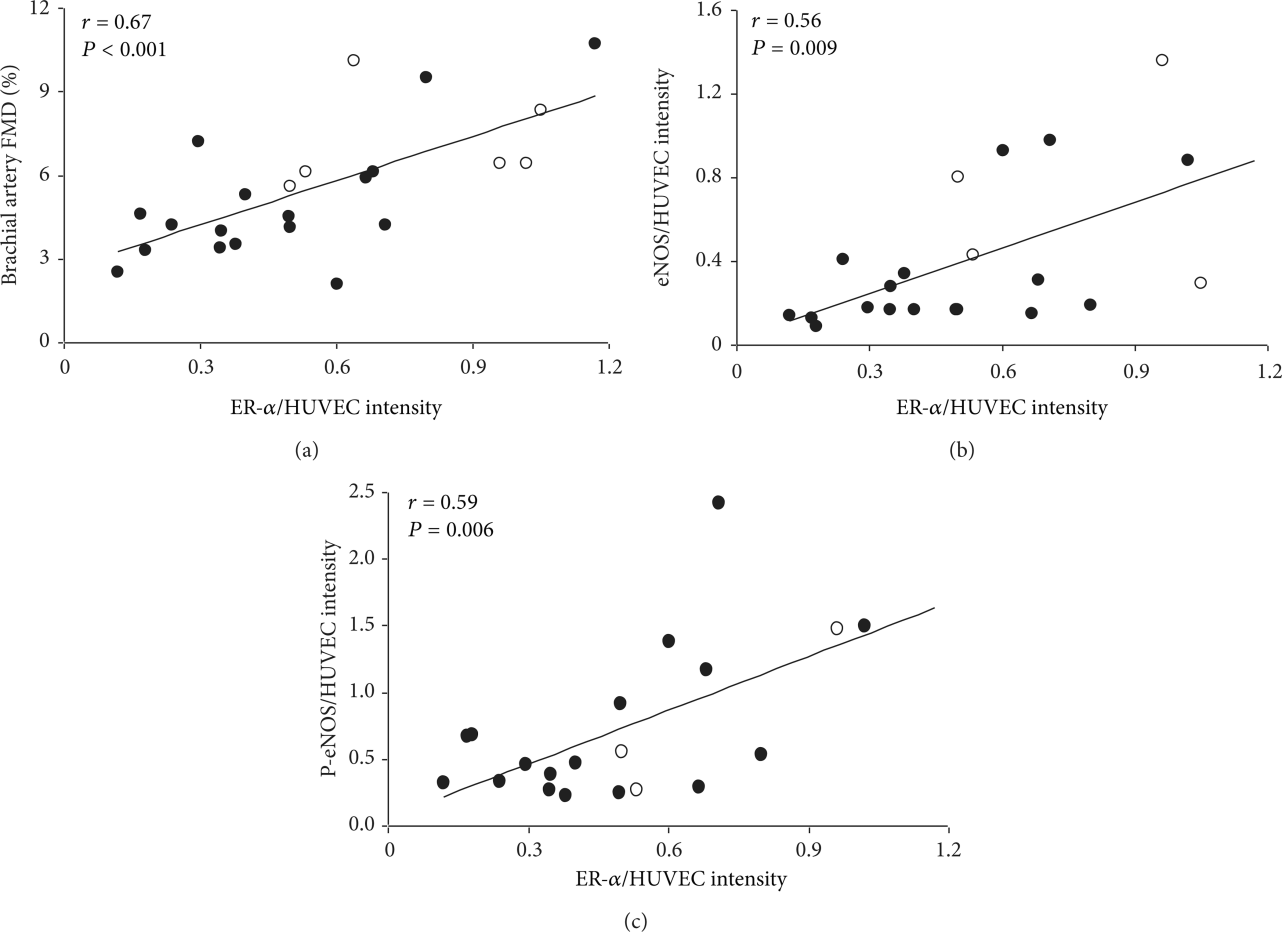
Endothelial ERα is positively correlated with (a) brachial artery EDV, (b) endothelial eNOS, and (c) endothelial phosphorylated-eNOS-Ser1177 (Pe-NOS) in premenopausal and postmenopausal women. Endothelial cells were harvested from peripheral veins of healthy premenopausal (open circles) and postmenopausal women (solid circles). Values are expressed as ratios to HUVEC controls. Adapted from Gavin et al. [4].

Results of studies in both experimental animals and in postmenopausal women are consistent with the notion that estrogen increases NO bioavailability. Indeed, plasma nitrate/nitrite concentrations (markers of NO production) and vasoconstrictor responses to acute blockade of eNOS are all augmented in the peripheral vasculature after, compared with before, estrogen exposure [17–19]. Estrogen triggers the release of NO through estrogen receptor (ER)α-mediated activation of eNOS as well as increased eNOS transcription [20, 21]. It has been reported that a functional ERα is important for NO bioavailability and normal vascular function, independent of circulating estrogen concentrations [22, 23]. Prolonged estrogen deficiency can lead to a marked reduction in ERα expression resulting in a functional impairment of ERα/eNOS signaling [24]. We previously demonstrated that ERα is reduced in endothelial cells harvested from peripheral veins of healthy estrogen-deficient postmenopausal compared with premenopausal women and that reduced ERα is associated with impaired EDV [4] (Figure 2). Moreover, *in vivo* endothelial ERα was positively correlated with eNOS protein and phosphorylated eNOS (Ser1177). These data suggest that ERα influences vascular endothelial function, in part by modulating eNOS and its state of activation. Whether vascular ERα expression and eNOS signaling are altered during the perimenopausal years warrants further investigation. Additionally, it would be important to determine whether targeting this pathway can preserve ERα expression and eNOS function during the menopause transition [25].

### 2 3. Oxidative Stress

A key mechanism contributing to the reduced endothelial dysfunction with estrogen deficiency and aging is oxidative stress [26–30]. Oxidative stress represents the imbalance between the production and destruction of reactive oxygen species (ROS). Overproduction of ROS can impair endothelial function by suppressing NO synthesis and scavenging NO, decreasing its overall bioavailability [31, 32]. Estrogen has direct antioxidant effects *in vitro* and *in vivo* and is thought to play an inhibitory role in either the production and/or scavenging of ROS [27, 28, 33]. For example, estrogen inhibits nicotinamide adenine dinucleotide phosphate (NADPH) oxidase, a major source of superoxide (O^−^) that reacts with NO to generate peroxynitrite (ONOO^−^) [26, 34]. In ovariectomized animals, estrogen treatment preserved EDV by protecting NO from scavenging by ROS [27, 28]. Consistent with this, we recently demonstrated that the impaired brachial artery EDV in estrogen-deficient post-menopausal women is improved with a systemic infusion of the antioxidant ascorbic acid, a well-described experimental model used to acutely reduce ROS [9]. Similarly, Virdis et al. demonstrated that the impaired resistance vessel EDV secondary to acute endogenous estrogen deficiency following oophorectomy is reversed with a local infusion of ascorbic acid [35]. However, there was no effect of ascorbic acid on EDV in women before oophorectomy, in healthy controls, or in oophorectomized women treated with estrogen for 3 months [35]. These observations support the idea that oxidative stress, due at least in part to the loss of the antioxidant effects of estrogen, is a major contributor to the endothelial dysfunction observed in postmenopausal women. Whether oxidative stress is mechanistically linked to the decline in endothelial function during the perimenopausal years in women is unknown.

### 2.4. eNOS Uncoupling

In vascular disease states characterized by oxidative stress, impaired EDV is thought to be partially mediated by eNOS uncoupling. Under physiological conditions, eNOS produces NO from the interaction with L-arginine. However, under pathophysiological conditions including aging (and possibly estrogen deficiency), eNOS can become uncoupled and produce ROS instead of NO. One mechanism that can lead to eNOS uncoupling is reduced bioavailability of tetrahydrobiopterin (BH_4_), an essential cofactor for normal eNOS function [36, 37]. BH_4_ can be limited when there is decreased biosynthesis from guanosine 5′-triphosphate (GTP) *via* the rate-limiting enzyme GTP cyclohydrolase I (GTPCH I) and/or *via* reduction of the inactive BH_2_ by dihydrofolate reductase (DHFR) [38]. Additionally, BH_4_ can be oxidized by peroxynitrite (ONOO^−^) and other oxidases (i.e., NADPH oxidase) [36, 37].

**Figure 3:**
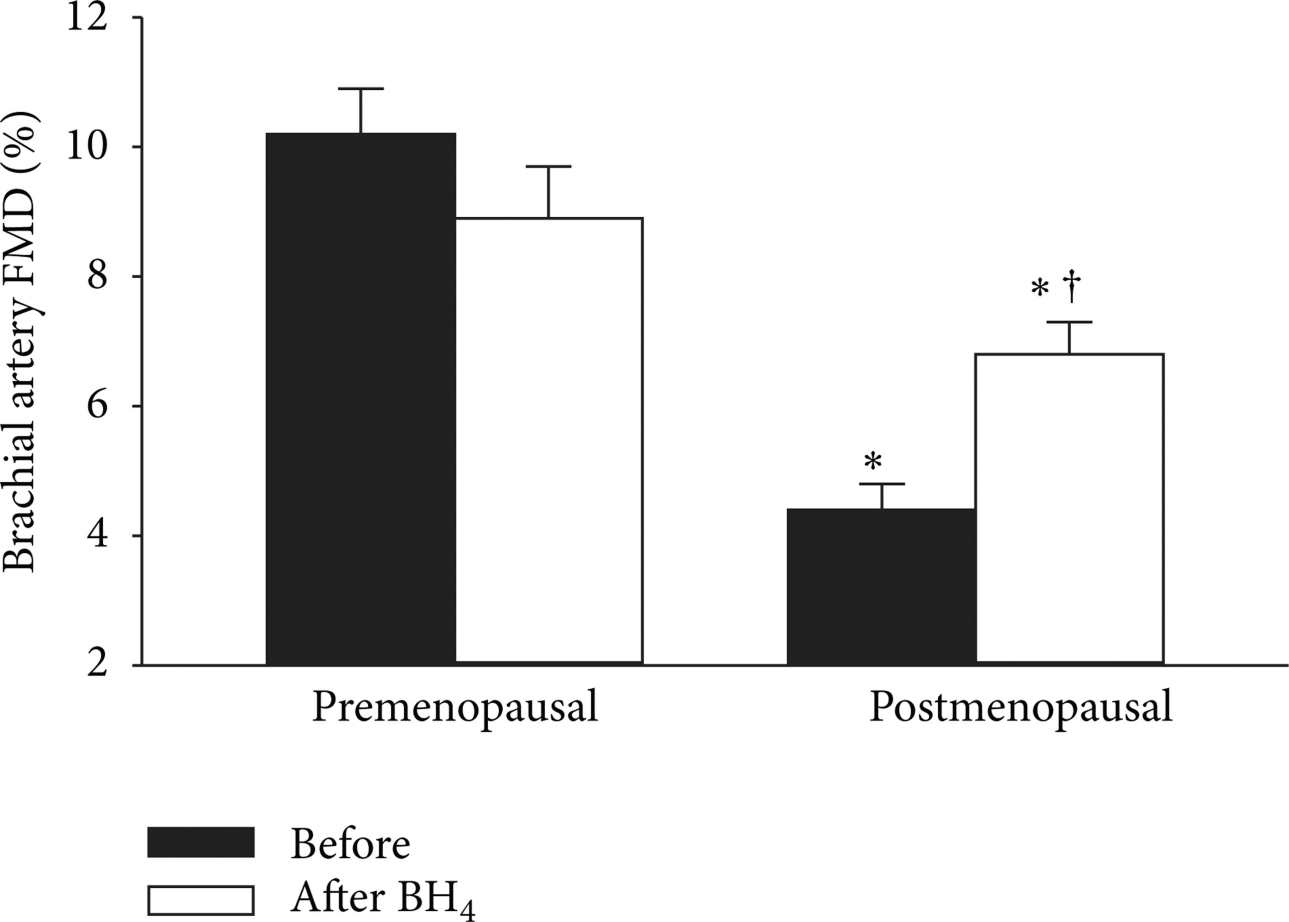
Brachial artery FMD before and after oral BH_4_ administration in premenopausal and postmenopausal women. * *P* < 0.0001 versus premenopausal women; ^†^*P* < 0.001 versus baseline of the same group. From Moreau et al. [5].

In ovariectomized animals, the impaired EDV is associated with reduced aortic BH_4_ and elevated ROS; treatment with estrogen or BH_4_ restores aortic BH_4_ content, decreases O^−^ production, and increases NO and EDV [33, 39]. In a recently completed investigation, we examined the role of BH_4_ and eNOS uncoupling in mediating endothelial dysfunction in estrogen-deficient postmenopausal women [5]. Brachial artery EDV was measured before and 3 hr after oral BH_4_ (10mg/kg) in postmenopausal women and premenopausal controls. BH_4_ administration improved EDV in postmenopausal women by 35% (Figure 3) but as expected had no effect on premenopausal women. Because we were also interested in determining whether endothelial benefits of estrogen were related to vascular BH_4_, oral BH_4_ was administered to postmenopausal women following random assignment to short-term (2d) transdermal estradiol or placebo. Brachial artery EDV increased in response to estrogen treatment; however, there was no further improvement with BH_4_ administration [5]. These findings suggest that at least part of the impairment in EDV in estrogen-deficient postmenopausal women is related to reduced vascular BH_4_ and eNOS uncoupling. However, because we did not measure BH_4_ in the blood or at the tissue level we can only speculate on this potential mechanism. Additionally, whether eNOS uncoupling begins to occur during the perimenopausal years warrants further study.

### 2.5. Tumor Necrosis Factor (TNF)-α and Inflammation

TNF-α is a pleiotropic inflammatory cytokine that plays a key role in in a variety of biological processes including endothelial activation and oxidative stress [40, 41]. As such, TNF-α and vascular inflammation are considered key mediators of vascular dysfunction with aging and estrogen deficiency. Indeed, ovariectomy in rats results in an upregulation of TNF-α and impaired endothelial function [40, 42]. TNF-α can stimulate the production of acute phase reactants (e.g., c-reactive protein (CRP), neutrophils) and can activate endothelial cells to express adhesion molecules and other chemoattractants and to promote leukocyte accumulation in the vascular wall [43]. Endothelial activation alters the functional phenotype of the endothelium from anti-inflammatory to proinfammatory, related in part to a decrease in the production and bioavailability of NO, which in itself is anti-inflammatory [44]. TNF-α can also decrease the bioavailability of NO by increasing the production of ROS and NO scavenging [40, 41, 45, 46], downregulating and inactivating eNOS [47], and decreasing BH_4_ and uncoupling eNOS [48]. Finally, TNF-α can upregulate other inflammatory markers (e.g., inducible NOS (iNOS) and vasoconstrictors) (e.g., ET-1, angiotensin II) [41, 42, 49].

In women, the decrease in ovarian function with menopause is associated with spontaneous increases in proinflammatory cytokines, which can be prevented with estrogen administration [50–54], suggesting that estrogen may also benefit the vasculature through anti-inflammatory effects. Estrogen antagonizes the proinflammatory effects of TNF-α by inhibiting nuclear factor-kappa-B (NK-κB), an inducible nuclear transcription factor [55]. Estrogen may also regulate inflammatory cytokines by enhancing NO release [56] and through its antioxidant effects, specifically by reducing ROS-stimulated proinflammatory cytokine expression [40]. We recently demonstrated that acute inhibition of TNF-α with the TNF blocker etanercept improves brachial artery EDV in estrogen-deficient postmenopausal women but has no effect on EDV in eumenorrheic premenopausal women or postmenopausal women treated with transdermal estradiol [14]. Collectively, the available evidence suggests that TNF-α is mechanistically involved in endothelial dysfunction with the loss of ovarian function, but whether TNF-α and inflammation are involved in the decline in endothelial function during the perimenopausal years warrants further study.

## 3. Large Elastic Arterial Stiffening

Arterial compliance reflects the ability of an artery to expand and recoil with cardiac pulsation and relaxation [57]. Through arterial distention, large elastic arteries in the cardiothoracic circulation (primarily the aorta and carotid artery) act to “buffer” the rise in systolic pressure by storing a portion of the ejected stroke volume during systole, while maintaining a continuous and steady blood flow across the capillary beds. Arterial compliance is primarily determined by the intrinsic elastic properties of the artery [58]. These structural elements include the endothelium, elastin and collagen composition of the intimal-medial wall, and the smooth muscle cells [59, 60]. Changes within these structural elements or the functioning of these components can result in a reduced “buffering capacity” or increased arterial stiffness.

**Figure 4:**
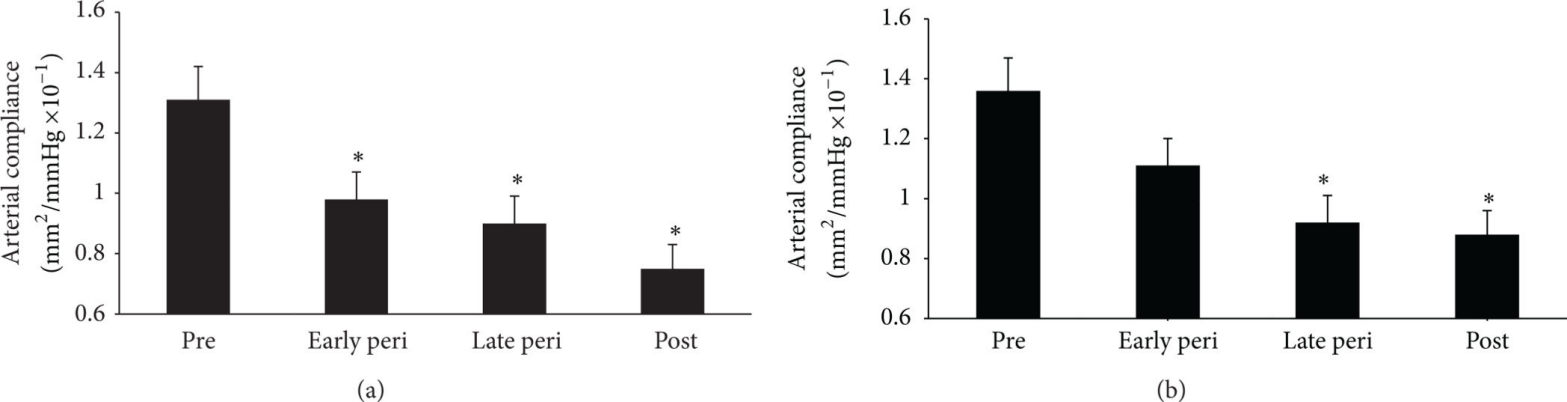
Carotid arterycompliance across the stages of the menopause transition. (a) Carotid artery compliance computed using brachial artery blood pressures and with (b) carotid pressures measured with applanation tonometry. * *P* < 0.05 versus premenopausal women. Adapted from Hildreth et al. [6].

In women and men, large elastic arteries stiffen (decreased arterial compliance) with advanced age even in the absence of clinical CVD [8, 61, 62]. However, in women the age-associated increase in large artery stiffness appears to be accelerated during the menopause transition presumably due to a decline in ovarian function and estrogen deficiency [61–63]. Indeed, we recently demonstrated that large elastic artery stiffening was progressively greater across the stages of menopause, beginning in early perimenopause [6] (Figure 4). Moreover, we [5, 8, 9] and others [62, 64–66] have previously shown that among postmenopausal women arterial stiffening is greater in non-HT users compared to chronic HT users and both acute and chronic estrogen treatment decrease arterial stiffening in postmenopausal women.

### 3.1. Mechanisms Underlying Arterial Stiffening across the Menopause Transition

The mechanisms that contribute to arterial stiffening of large elastic arteries across the menopause transition are not completely understood but likely involve structural changes within the arterial and functional changes of vascular smooth muscle cell tone. Both of these components are discussed below.

### 3.2. Structural Alterations

**Figure 5:**
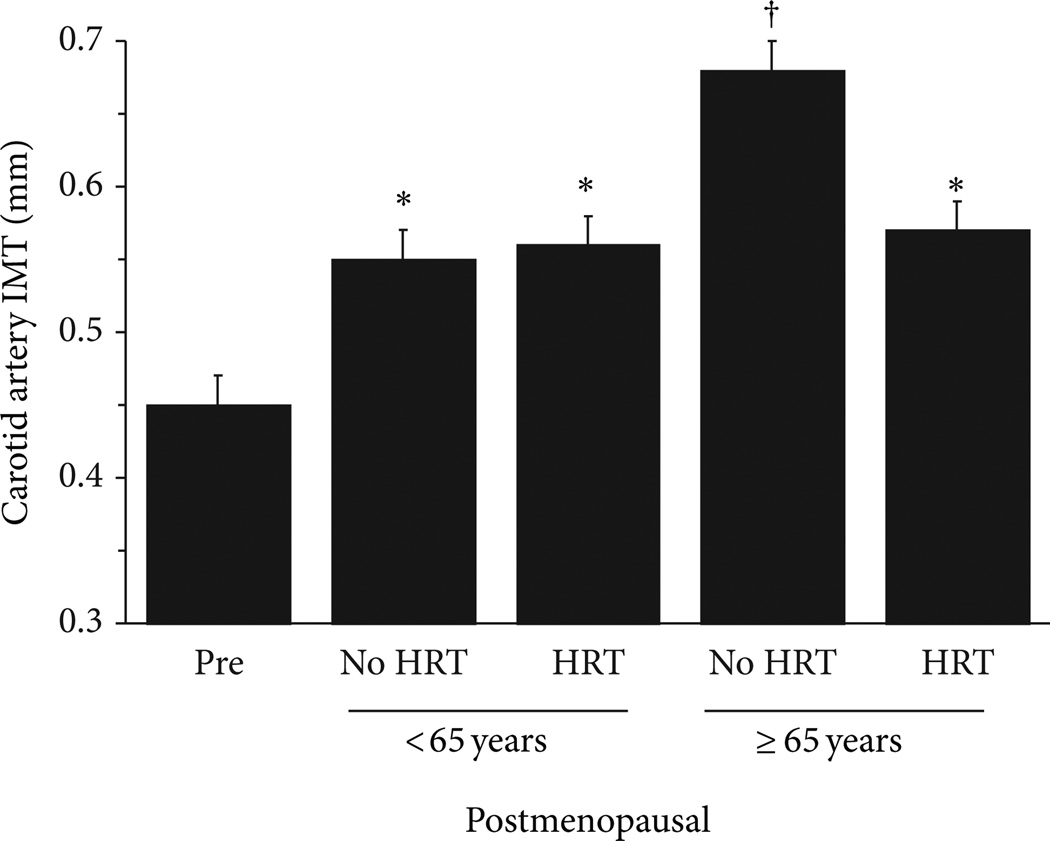
Carotid artery IMT in postmenopausal women <65 and ≥65 years who are chronic hormone therapy users (HRT) or non-HRT users and premenopausal controls (Pre). * *P* < 0.0001 versus premenopausal women; ^†^ *P* < 0.05 versus ≥65 years no HRT. Adapted from Moreau et al. [7].

Age-associated increased arterial stiffening has been attributed to structural changes within the arterial wall including increased collagen and reduced and fragmented elastin content [2, 67] and increased intimalmedial thickness (IMT) [7, 68]. Animal studies demonstrate that estrogen can increase elastin content, inhibit collagen synthesis [69, 70], and prevent smooth muscle cell proliferation into the intimal space [71, 72], potentially countering the structural changes associated with arterial stiffening. Indeed, we and others have demonstrated that long-term HT initiated around the menopause may attenuate IMT, primarily in the latter phase of the menopausal years [7, 73–76] (Figure 5). Collectively, these data suggest that estrogen has a modulatory influence on structural alterations that are observed with advancing age.

### 3.3. Functional Changes in Smooth Muscle Cell Tone

Functional changes in the contractility or tone of the artery also likely contribute to large elastic artery stiffening across the stages of menopause transition. The arterial wall is composed of vascular smooth muscle cells which vasodilate and vaso-constrict in response to a number of circulating and local vasoactive mediators including those released by the vascular endothelium, as well as estrogen. One endothelial-derived substance, NO, is a key regulator of arterial stiffness [77, 78]. We have reported a strong correlation between basal brachial artery EDV and carotid artery compliance in premenopausal and postmenopausal women and between improvements in both of these measures in response to estrogen [5]. These data support the idea that large elastic arterial stiffening in estrogen-deficient postmenopausal women is mediated, in part, by an elevated state of vascular smooth muscle cell vasoconstrictor tone, possibly related to reduced NO bioavailability.

### 3.4. Oxidative Stress

**Figure 6:**
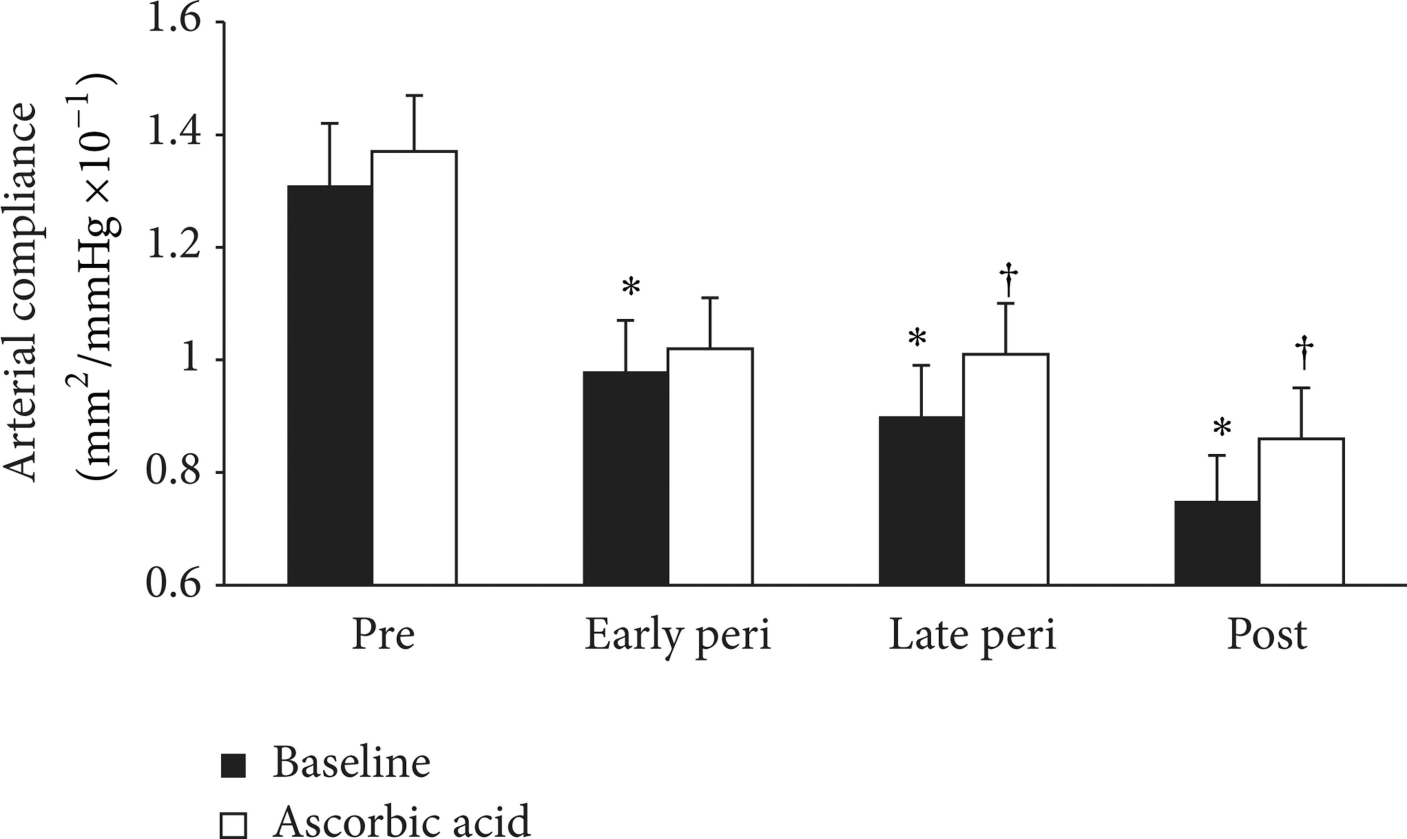
Carotid artery compliance during systemic saline (control; black bars) and ascorbic acid (antioxidant; white bars) infusions in premenopausal (pre), early perimenopausal (peri), late perimenopausal, and postmenopausal (post) women. * *P* < 0.05 versus premenopausal women. ^†^ *P* < 0.05 versus saline of the same group. Adapted from Hildreth et al. [6].

As mentioned earlier, estrogen is a potent endogenous antioxidant and can influence the balance of oxidant and antioxidant forces and prevent the scavenging of NO by ROS. Similar to our previous investigation on endothelial function [9], we also show that carotid artery compliance increases (arterial stiffness decreases) during the systemic infusion of ascorbic acid in estrogen-deficient postmenopausal women but remains unchanged in premenopausal women, indicating that oxidative stress contributes to large artery stiffening in estrogen-deficient postmenopausal women. We recently extended this investigation to determine whether arterial stiffening across the menopause transition is related to a shift in the oxidant/antioxidant balance toward oxidative stress. We found that the ascorbic acid infusion increased carotid artery compliance in late perimenopausal and postmenopausal women but not in early perimenopausal or premenopausal women (Figure 6) [6]. These findings suggest that effects of oxidative stress on arterial stiffening manifest with declines in ovarian function and estradiol concentrations that coincide with the late perimenopausal and postmenopausal years. It is also possible that adverse changes in metabolic risk factors that occur during the late perimenopausal and postmenopausal period, and that are known mediators of oxidative stress [79–81], may amplify oxidative stress and arterial stiffening in women.

It is important to note that the ascorbic acid infusion did not restore carotid artery compliance in perimenopausal or postmenopausal women to premenopausal levels. This suggests that ascorbic acid did not completely suppress ROS or that other sources of ROS not scavenged by ascorbic acid are involved and/or that other mechanisms independent of ROS are involved. Although ascorbic acid is a potent scavenger of superoxide and other ROS, it is a relatively weak scavenger of ONOO^−^ [82], a strong oxidant that can damage lipids, mitochondria, protein, and DNA leading to cell death. Peroxynitrite also oxidizes BH_4_ causing eNOS uncoupling, reduced NO, and further ROS production. Even though ascorbic acid can stabilize eNOS by recycling oxidized BH_4_, BH_4_ reacts with ONOO^−^ 6–10 times faster than ascorbic acid [82]. Thus, ascorbic acid may not have improved arterial stiffness in early perimenopausal women or fully restored it in late perimenopausal and postmenopausal women because ascorbic acid did not fully protect BH_4_ from oxidation by ONOO^−^. In this regard, we recently demonstrated that administration of BH_4_ increases carotid artery compliance in estrogen-deficient postmenopausal women, supporting the idea that BH_4_ deficiency and eNOS uncoupling are mechanistically linked to the arterial stiffening in estrogen-deficient postmenopausal women [5]. Similar to ascorbic acid, BH_4_ supplementation did not restore carotid artery compliance to premenopausal levels indicating that other mechanisms are likely involved in arterial stiffening across the stages of the menopause transition.

Like endothelial dysfunction, vascular inflammation is a key modulator of arterial stiffness. Arterial stiffness is higher in chronic inflammatory disorders, such as rheumatoid arthritis [83, 84], and treating such patients with anti-TNF-α therapy decreases arterial stiffening [84]. Data from the Study of Women Across the Nation (SWAN) Heart reported that the association between CRP and pulse-wave-velocity (PWV), a marker of arterial stiffness, in women undergoing the menopause transition was modified by menopause status. Specifically, the association between CRP and PWV was stronger in late perimenopausal and postmenopausal women compared to premenopausal and early perimenopausal women, suggesting that inflammation may contribute to arterial stiffening more strongly among women during the late perimenopausal and postmenopausal periods [85]. Consistent with this, we recently demonstrated that acute anti-TNF-α treatment with etanercept increased carotid artery compliance in estrogen-deficient postmenopausal women [14]. Additionally, whereas short-term transdermal estradiol increased carotid artery compliance in previously estrogen-deficient postmenopausal women, coadministration of etanercept with estradiol did not further augment carotid artery compliance suggesting that estradiol may alter proinflammatory cytokine effects.

## 4. Endurance Exercise Training Effects on Vascular Aging in Women

**Figure 7:**
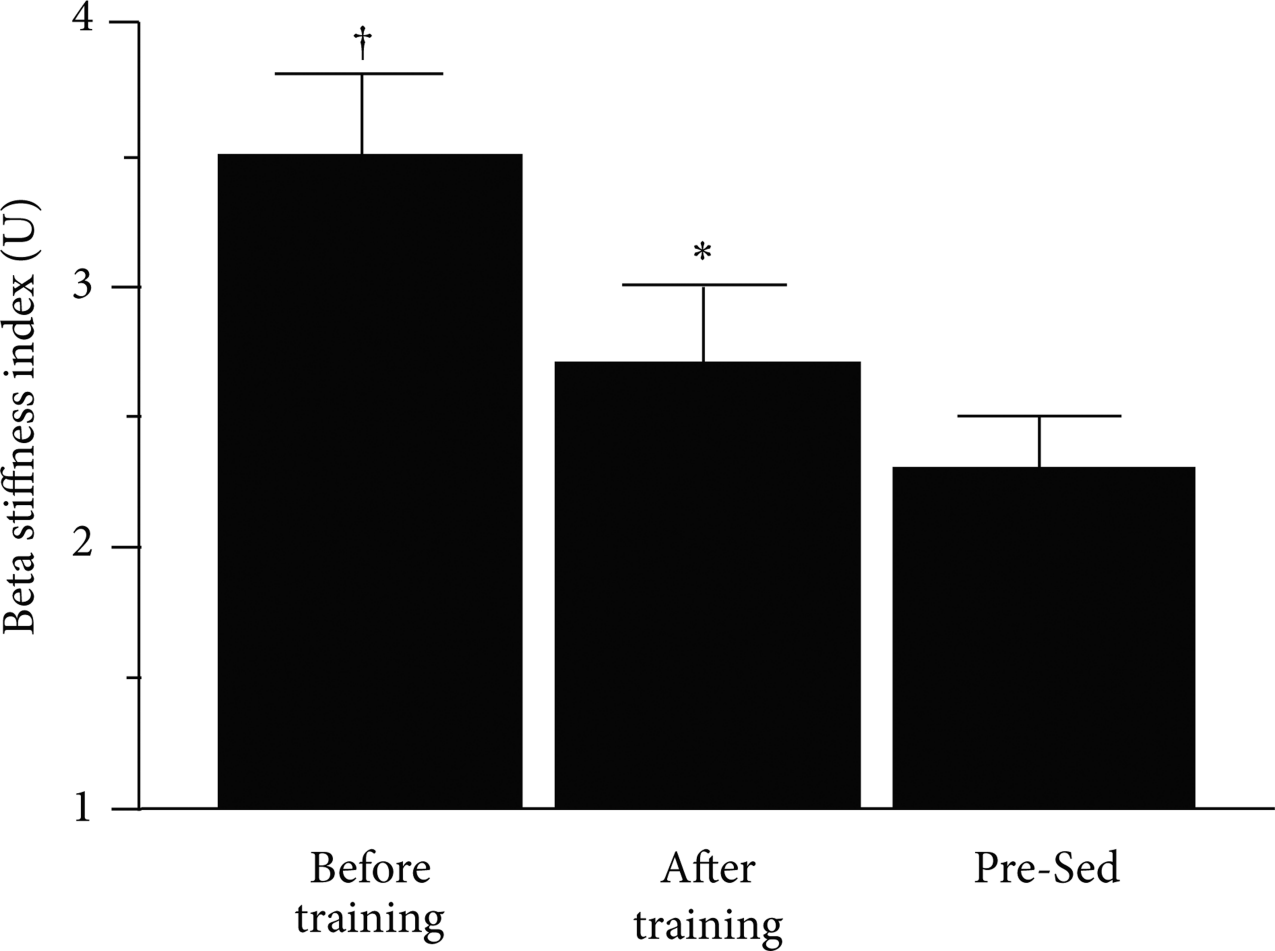
Carotid artery beta-stiffness index before and after a 3-month endurance exercise training intervention in previously sedentary postmenopausal women who were chronically taking hormone replacement therapy and in premenopausal sedentary controls. * *P* < 0.05 versus before training; † versus Premenopausal Sedentary Controls. Adapted from Moreau et al. [8].

Regular physical activity is associated with a reduced risk of CVD related events and is promoted as a therapeutic strategy for delaying and/or improving vascular aging [86–88]. In general, regular habitual exercise can attenuate vascular aging in women but this is not realized across all biomarkers of vascular aging. For example, most studies show that habitually endurance-trained postmenopausal women have reduced arterial stiffening compared to age-matched sedentary postmenopausal women [8, 89, 90] and that endurance exercise training programs can decrease arterial stiffness in previously sedentary postmenopausal women [8, 91–93] (Figure 7).

**Figure 8:**
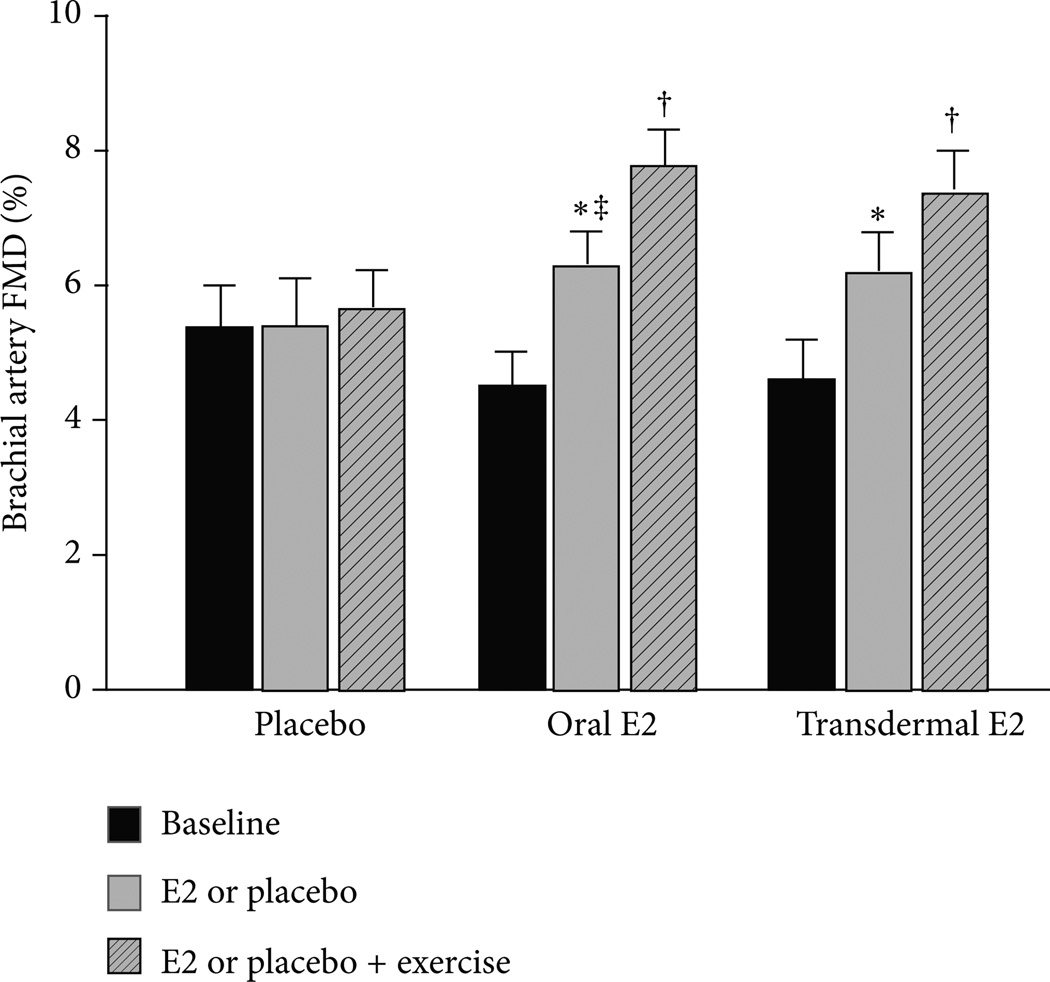
Brachial artery flow-mediated dilation (FMD) before and after 12 weeks of oral or transdermal estradiol or placebo treatment and an additional 12 weeks of estradiol or placebo treatment plus aerobic exercise training. * *P* < 0.01 versus baseline; ^†^ *P* < 0.01 versus 12 weeks; ^‡^ *P* < 0.01 versus placebo 12 weeks. Reproduced from Moreau et al. [9].

In contrast, beneficial adaptions to endurance exercise training on endothelial function appear to be diminished or absent in postmenopausal women compared to age-matched men [29, 94–100]. The reasons for this are unclear but may be related to differential exposure to sex hormones [101]. Unlike women whose endogenous estrogen concentrations undergo an abrupt decrease with menopause, a parallel change is not observed in men [102, 103]. In this regard, we recently investigated whether the attenuated adaptation of endothelial function to endurance exercise training in postmenopausal women is related, in part, to an absence of estrogen. We found that brachial artery EDV increased in response to 12 weeks of moderate intensity endurance exercise training (i.e., brisk walking) in previously sedentary postmenopausal women who were treated with either oral or transdermal estradiol but not in placebo treated women (Figure 8) [9].

Although some studies have reported a dose response to exercise in which higher intensity and/or volume of exercise was associated with better health outcomes, these studies have primarily been conducted in men [104, 105] or in populations with risk factors [106] or known CVD [107]. In healthy postmenopausal women not currently using HRT, improvements in brachial artery FMD were observed after 12 weeks of mixed moderate to high intensity endurance exercise, although the improvement was seen only in those participants with greater baseline impairment [108]. The best evidence to date to address the issue of exercise dose has been most directly addressed by Pierce et al., who compared brachial artery FMD between sedentary and highly endurance-exercise-trained estrogen-deficient postmenopausal women and middle-aged and older men in a large cross-sectional study design (*n* = 167) [99]. Endurance trained women and men had been engaged in habitual vigorous aerobic exercise ≥5 days/week, >45 minutes/day for at least the past 5 years. In habitual endurance-trained men, brachial artery FMD was 50% greater than their age-matched sedentary peers, whereas in postmenopausal women there was no difference between endurance-trained and sedentary women. That FMD was not different between these two groups, and the fact that the endurance-trained postmenopausal women had a similar maximal aerobic capacity as men argues against the notion that a higher or more sustained exercise stimulus may be needed to improve FMD in women. Moreover, in the same study, an 8-week moderate intensity exercise program improved FMD in previously sedentary middle-aged and older men to levels similar to those observed in the habitually endurance-trained men, but again no improvement was observed in previously sedentary postmenopausal women who participated in the same exercise training program [99]. Similar to the Pierce et al. study, our work demonstrated that a control group of estrogen-deficient postmenopausal women who habitually performed vigorous endurance exercise (i.e., running) brachial artery FMD was similar to that of the intervention group at baseline and to that of the placebo group after the exercise intervention [9].

These findings support the idea that estrogen is necessary to reap the beneficial effects of endurance exercise training on endothelial function in women. Indeed, brachial artery EDV is reduced in highly trained amenorrheic premenopausal athletes compared to eumenorrheic athletes and sedentary eumenorrheic controls and is restored after recovery of the menstrual cycle and with oral contraceptives [109, 110]. We also previously demonstrated that moderate intensity endurance exercise training restores carotid artery compliance in postmenopausal women who were chronic hormone therapy users [8]. Collectively, these data indicate that estrogen status plays a key permissive role in the adaptive response of the vasculature to endurance exercise training in women. Future studies should further examine the role of estrogen status in response to exercise training, particularly during the perimenopausal period.

## 5. Summary and Conclusions

**Figure 9:**
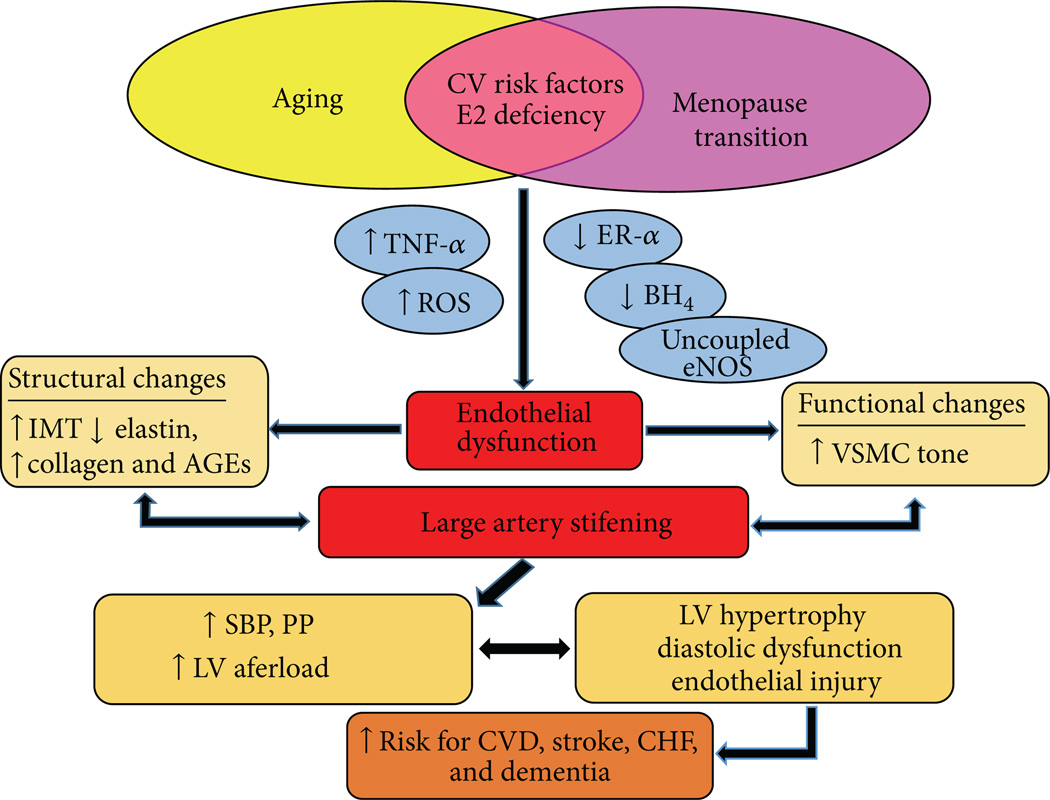
Working model of vascular aging across the menopause transition in healthy women.

CVD is a major public health concern for women [111], and vascular aging is a major risk factor involved in the etiology of CVD [2]. In women, the menopause transition may be a triggering event that leads to increased vascular vulnerability and accelerated vascular aging due to changes in the hormonal environment. As such, a further understanding of the biological defects mediating vascular aging in women as they transition through menopause and lose the protective effects of estrogen on the vasculature is needed. One of the most important areas in which additional research is needed is elucidating the underlying mechanisms that contribute to vascular aging in women across the menopause transition. Our research suggests that oxidative stress, vascular inflammation, ERα, and eNOS dysfunction contribute to an acceleration in vascular aging with estrogen deficiency in women (Figure 9). However, there are potentially many other mechanisms not discussed in this review that could be involved. For example, animal studies demonstrate that the local renin-angiotensin system and the endothelin system play important roles in the pathogenesis of EDV with estrogen deficiency and aging [2, 112]. Research into these mechanisms and others, where they occur across the stages of the menopause transition, and whether they are triggered by estrogen deficiency will help to inform the critical window of intervention for the maintenance of endothelial function and prevention of future CVD in women. If vascular aging is accelerated with declines in ovarian hormone levels with the menopause transition, this would have important implications for aggressively promoting intervention strategies for CVD prevention in women during the perimenopausal years. Because life expectancy is increasing, women will be spending more than a third of their lifespan after menopause. Thus, it is of clinical importance to apply evidence-based therapeutic strategies for CVD prevention in women. The negative findings from the Women’s Health Initiative (WHI) study have shifted clinical practice from treating menopausal women with HT to recommending lifestyle interventions including exercise for CVD prevention. Although exercise may be an effective strategy in men, it may be less advantageous in women, particularly for preserving or improving endothelial function. Thus, another important question for future study is determining what type, volume, and intensity of exercise may optimize vascular health in women. Current exercise recommendations may need to be modified to consider the hormonal state of the woman in order to fully realize the benefits of exercise. Nonetheless, in order to inform treatment or effective intervention strategies, understanding the mechanistic defects underlying vascular aging and the diminished responsiveness of the vasculature to exercise in older women will provide the foundation for future research to determine effective sex-specific therapeutic strategies for preserving vascular function as women age.
